# Polish Nurses’ Attitude to e-Health Solutions and Self-Assessment of Their IT Competence

**DOI:** 10.3390/jcm10204799

**Published:** 2021-10-19

**Authors:** Anna Bartosiewicz, Joanna Burzyńska, Paweł Januszewicz

**Affiliations:** Institute of Health Sciences, Medical College of Rzeszow University, 35-959 Rzeszów, Poland; j-burzynska@wp.pl (J.B.); PJanuszewicz@polsat.com.pl (P.J.)

**Keywords:** e-health, healthcare digitalization, IT competence, tele-nursing

## Abstract

In many countries, the implementation and dissemination of e-services for healthcare systems are important aspects of projects and strategies, as they contribute to significantly improving the access to such a system. The aim of the study is to analyze nurses’ opinions on the application of the e-health solutions at work and the self-assessment of their IT competence. A linear stepwise regression allowed for the visualization of independent variables significantly influencing considerably the level of IT competency. Reduced IT competency was found in the group of nurses who rated the impact of the Internet and the new technologies as lower on the health care and general lives of modern people (β = 0.203; *p* < 0.0001), recommended e-health solutions to a lesser extent (β = 0.175; *p* < 0.0001), rated e-health solutions lower in relation to the patient (β = 0.149; *p* < 0.0001), and were older in age (β = 0.095; *p* = 0.0032). IT competence has become an indispensable requirement for nurses in fulfilling their professional roles. The quality of using new technologies in the work of nurses depends on their IT competence.

## 1. Introduction

The development of information and communication technologies has had a huge impact on the functioning of modern society in many areas. The Internet is no longer just a tool for accessing information, but one of the basic forms of multi-tasking [1,2]. The system of healthcare makes extensive use of technological innovations, creating opportunities for medical staff, including nurses, to care for their patients in the best possible way. Even if electronic communication is considered to have diminished the chance for personal contact between a patient and a nurse, it has also become a way to improve the level of care, especially in difficult and critical situations.

In an increasingly computerized society, e-health is a dynamically developing area of health protection that is bringing tangible benefits. The integration of IT competence and its relationship with providing e-health services is a priority in many countries around the world, including Poland [3]. The development and implementation of e-health strategies are considered as a priority in the Digital Agenda for Europe to enable patients and health care professionals to apply information technology to maintain and improve their health [1,3]. The process of digitization in the health care system of Poland was one of the main areas of the strategy for the development of nursing and obstetrics in Poland, which was presented on 9 January 2018 by the Ministry of Health as a system solution and support for nursing care [4]. Both the implementation and dissemination of the e-services in the system of health care are important aspects of health care projects and strategies in many countries [5]. The problem that many countries will have to face in the near future, resulting in limiting significantly the access to the professional care, is the shortage of nursing staff and the average age of working nurses, that is 50–53 years [6]. According to the World Health Organization (WHO), the number of 7.3 million nursing personnel worldwide is currently deficient in relation to the reported needs. In 2035 there will be a shortage of 12.9 million nurses, according to the report of the Third General Forum of Human Resources in Healthcare [6,7]. Global problems that have a huge impact on the health policy and the system of health care include demographic changes in societies, the steady increase in the number of the elderly people and the situation resulting from the ongoing COVID-19 pandemic. The question of long-term care is becoming a strategic area creating a high demand for care and nursing services [8,9]. The use of new technologies in patient care is one of the ways to solve the problem of staff shortages in the system of healthcare, as shown by tele-nursing services having been used in the USA, Japan, Australia, and the Scandinavian countries for many years [10,11,12,13,14]. The development of the e-nursing has proved to be not only a benefit, but a necessity especially during the ongoing COVID-19 pandemic and the need to provide remote care to patients facing staff shortages, which the ongoing pandemic has only exacerbated [15,16]. The pandemic has become a sort of catalyst for the process of digitization in the health sector around the world [17,18,19].

The rationale of the research undertaken was twofold. On the one hand, the progressive character of the process of digitization of the health sector was noticeable even before the COVID-19 pandemic, with actions indicating that the implementation of the e-health services was becoming a priority of many countries. On the other hand, there is a noticeable lack of readiness as well as competency on the part of professionally active nurses to accept and implement the more modern solutions in practice. The main aim of this study is to analyze the nurses’ opinions on the application of the e-health solutions at work and the self-assessment of their IT competence.

## 2. Materials and Methods

### 2.1. Study Design and Participants

This is a cross-sectional, descriptive study. The study was conducted at the turn of 2019 and 2020 among nurses working in Primary Healthcare (PHC), hospital (H), private sector (PS) and Outpatient Specialist Care (OSC) in the South-Eastern part of Poland. Invitations were sent to 34 medical entities randomly selected via a randomized algorithm program. The sample size was determined with the help of the EPI INFO (StatCalc) software. A multistage random cluster sampling method was used. The message contained data on the planned study, and 12 invited medical entitles gave positive feedback. The following criteria for the selection respondents were adopted: professionally active nurses, having the right to practice, working in PHC, OSC, PS and H, willing to participate in the study. Nurses working at the institutions that agreed to participate in the study were fully informed in writing and verbally about the nature of the study. They were assured of the voluntary participation in the survey and the anonymity of the answers provided. The survey questionnaires along with the consent form were delivered to the facilities. To ensure the confidentiality of data, the questionnaires were numbered and return in sealed envelopes attached to the questionnaires, the correct completion of which was equivalent to the nurses expressing their participation in the study. A completed questionnaire automatically constituted consent to participate in the study. Ultimately, 1470 questionnaires were distributed, 860 (58.5%) were collected back and 11 were rejected due to incomplete responses. Data from 849 questionnaires were put on an encoded excel sheet and subjected to statistical analysis. Finally, the study group included nurses working in primary health care (N = 395), hospitals (N = 307), private sector (N = 80) and outpatient specialist care N = 71).

**Figure 1:**
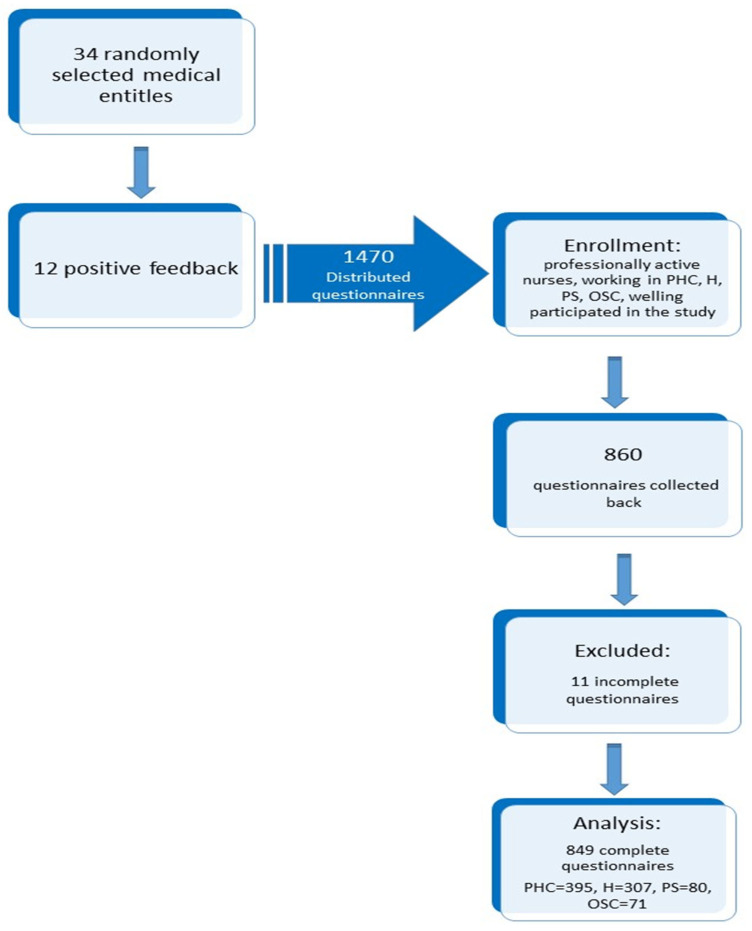
Flow chart demonstrating the selection of the study participants.

Nurses participating in the study constituted a representative group for all nurses working in this region (the error threshold was 5%, i.e., the test power was 0.95). All participants of the study were informed about the possibility of withdrawing from the study at any stage without any consequences (Figure 1).

### 2.2. Questionnaire

The study used the authors’ questionnaire. The survey questions concerned the socio-demographic data of the surveyed nurses (age, education, additional qualification, and workplace).

The survey questionnaire used in the study took into account the following issues:

- The section on the frequency of using electronic devices such as: a computer, tablet, smartphone, e-mail address and mobile applications by nurses.
- The “IT competence” section concerned the assessment of one’s ability to use electronic device.
- The section of “Recommendation of the E-health solutions” concerned the recommendation of electronic solutions for patients.
- The section of “Assessment of the e-health solutions” concerned the benefits of the using e-health solutions in everyday work.

The statements about the feelings of the surveyed nurses about the presence of the new technologies in the everyday lives of modern people (File S1: questionnaire template as Supplementary Material attached).

### 2.3. Statistical Analysis

The estimation method and the following statistical methods were used: in order to present the data, the method of descriptive statistics was used—arithmetic mean (M), the value of which determines the average level of a given variable, and standard deviation (SD), a statistical measure of scattering the results around the expected value. Factor analysis used the principal components method, which allowed for the creation of coherent indicators assessing IT competency, assessing the impact of the Internet on modern human life, recommendations for e-health solutions and evaluation of e-health solutions. The reliability of the obtained indicators was also verified with the Cronbach’s alpha coefficient. The stepwise linear regression assessed the impact of selected variables on the level of IT competence, calculated generally for the entire study group and separately for separate subgroups of nurses.

The normality test for the general model is R = 0.428, R^2^ = 0.183.

A significance level of *p* < 0.05 was assumed.

Calculations were performed with the IBM SPSS program Statistics 20 (IMB, Armonk, NY, USA).

### 2.4. Ethics

The study was approved by the institutional Bioethics Committee of the Rzeszow University (Resolution No. 6/12/2019) and all relevant administrative bodies.

## 3. Results

### 3.1. The Reliability and Validity of the Questionnaire

The Cronbach’s alpha and PCA were calculated for each section:

- Nurses’ IT competence

PCA: two components (called “Skills” and Need for Training) explained 52.72% of the variance, Cronbach’s alpha 0.457.

- Assessment of the influence of the Internet and new technologies on health care and modern life

PCA: one component, explaining 62.01% of the variance. Cronbach’s alpha 0.379.

- Recommendation of e-health solutions

PCA: two components explaining a total of 49.20% of the variance, Cronbach’s alpha 0.852.

- Assessment of e-health solutions

PCA: the two components (patient-per-solution, facility-based solution) accounted for a total of 61.86% of the variance, Cronbach’s alpha 0.894.

Additionally, intraclass correlation coefficient (ICC) was calculated (Table 1).

### 3.2. Characteristics of the Study Group

In the study 849 nurses participated aged 22 to 68. The mean age of the respondents was 41.05 ± 12.25 years. Half of the respondents were under 42 years old, 25% of the respondents were under 28 years of age, and 25% of the respondents were at least 51 years old. The majority of respondents were nurses with a BA (N = 341; 40.2%) and secondary medical education (N = 276; 32.5%). Only 101 nurses (11.9%) had additional qualifications. The study group consisted mainly of nurses working in PHC (N = 395; 46.5%) and in a hospital (N = 307; 36.2%), as well as nurses working in private sector (N = 80; 9%) and outpatient specialist care (OSC) (N = 71; 8.4%).

### 3.3. Findings

The nurses declared that they mainly use the Internet mainly several times a day (47.7%) or every day (41.7%). In private life, the respondents often mainly used a smart phone (81.7%) and a computer (62.9%). In professional work, a computer was used mainly (72.3%) (Table 2).

### 3.4. IT Competence

Nurses asked about their self-assessment of using IT devices/solutions and the Internet most often assessed their skills as good, sufficient and very good. When asked if they felt prepared to handle e-health solutions related to their professional work, nurses declared that they would “rather yes” and “yes”. Most of the respondents would benefit from additional training/courses in the field of shaping digital competence. In the opinion of the majority of nurses, today’s nursing education definitely keeps up with the digital challenges of the 21st century, but they also agreed with the opinion that the nursing program me should contain more learning outcomes to prepare for the acquisition of IT competency (Table 3).

### 3.5. Recommendation of e-Health Solution

The results showed that the surveyed nurses most often recommend obtaining laboratory test results and arranging medical appointments via the Internet in their daily work. If they had the opportunity, they would recommend the use of a mobile application constituting a knowledge base on health-related topics, applications reminding about the need to take medication and a video consultation with a doctor/nurse/midwife. Every fourth surveyed nurse believes that video consultations should not be recommended to patients, and every fifth did not recommend the use of mobile applications facilitating the analysis of tests (Table 4).

### 3.6. Assessment of e-Health Solutions

According to the surveyed nurses, the most useful e-health solutions are possibility to write out electronic sick leaves (46.5%), referrals (41.5%), easy and quick access to patients’ medical records (40.2%) and possibility to write electronic prescriptions (39.1%) (Table 5).

According to the majority of the surveyed nurses, the functioning of the health care system in the near future will be very dependent on new technologies and the Internet. The vast majority of nurses believe that the use of new technologies in everyday life and at work is very helpful (Table 6).

The step linear regression model (Table 7 and Table 8) showed that significant (*p* < 0.05) variables influence the nurses’ IT competencies in terms of skills and in term the need for training. The nurses’ IT competence was analyzed in two aspects: IT competencies as skills and IT competence as the need for training, which were influenced by quantitative variables: assessment of the influence of the Internet and new technologies on health care and lives of modern people, recommendation of e-health solutions, assessment of e-health solutions and age. The impact of these variables was assessed in total and broken down into education (three groups), specialization (two groups) and place of work (two groups: hospital and primary health care). In relation to IT competence analyzed as skills, higher scores mean lower skills.

Nurses’ IT competence in relation the need for training—higher results mean a higher need for training (Supplementary File S2: graphical presentation results as Supplementary Material attached.

## 4. Discussion

The aim of the study was to find out about nurses’ opinions on the application and use of the e-health solutions in their workplace and to assess their IT competence. The development of digital technologies is increasingly influencing the work of nurses around the world. A very good example of this is the growing number of various usages of electronic devices and the Internet, dependence on many tele-care models, robotic systems, and the growing presence of artificial intelligence in nursing [20,21,22,23]. Our results showed that the vast majority of nurses use the Internet (93%). In private life, the most frequently used electronic device is a smartphone (81.7%) and a computer (72.3%) at work. For many years, the health sector in many countries has been undergoing the process of digitization, and tele-medicine and tele-nursing successfully play a strategic role in many areas of patient care [24,25]. The nurses as a profession are more and more visible on the web, not only using electronic devices, providing services to their patients, but also participating in training, conferences, and internet fora. In this way, they build their image, learn, exchange experience, and provide each other with support. Social media are not only used to passively receive the information, but the virtual reality also helps to create reality, motivating to new challenges and lifelong learning. Similar results were obtained by the authors of a cross-sectional study conducted among 658 Chinese nurses who were social media users, and the vast majority (84.5%) of them believed that social media positively influenced their professional development and clinical practice [26]. According to Lefebvre et al. the use of social media is common among nurses, but the attitudes and perceptions of social media in this professional group differ greatly [27].

The obtained results concerning the nurses’ self-assessment regarding the use of electronic devices, IT solutions at work and the Internet showed that the respondents most often assessed their skills as good, sufficient or very good (35.5%, 29.0%, 27.8%). A study on the self-assessment of IT competence among almost three thousand Canadian nurses showed that the declared skills were mainly related to basic computer skills, while those related to information and knowledge management were at a low level. Similar results are presented by the Korean study; despite the fact that nurses are favourable to computerization and the use of electronic devices in their work, more than two-thirds of nurses (69.2%) stated that their general IT competence are below average. They obtained the highest results in the areas of IT related to security and confidentiality, and the lowest in tele-health. More than half of the respondents (58.9%) assumed that their computer skills are below average. The authors of the study suggest that improving basic computer skills and incorporating computer science into formal nursing programs are necessary to improve nurses’ competence in managing and using health information [27,28,29]. Moreover, although the nurses in our study mostly declared that they were prepared to handle e-health solutions related to their professional work (71.8%) (28.3%), the majority of the respondents (79.2%) would benefit from additional training/courses in the field of shaping digital competence. Although they believe that today’s nursing education definitely keeps up with digital challenges (55.3%), they believe that the nursing programme should better prepare nurses to acquire IT competence (63.6%). According to Booth, there is an urgent need to create undergraduate and graduate education opportunities for nurses in computer science, digital health and data processing. Moreover, the author emphasizes that nurses should be able to learn from computer scientists and engineers in order to be able to fully lead the development of new models of patient care and take advantage of the opportunities created by digital technologies [30]. Despite significant progress to date, challenges remain with the use of digital technology by the nurses. The age-old problem is that nurses are generally not keeping up with the rapid changes in digital technologies and their impact on the society. This in turns limits the potential benefits of nursing practice in patient care. Many studies indicate the need for training nurses in this field [31,32,33]. The authors of the systematic review emphasize that regularity of such training, as well as motivation and support for nurses acquiring IT competence, is very important [34].

The study showed that the surveyed nurses most often recommend obtaining laboratory test results via the Internet (45.9%) and arranging medical appointments via the Internet (41.5%) in their daily work. If they had the opportunity, they would recommend first of all the use of a mobile application constituting a knowledge base on health-related topics (59.7%), applications reminding about the need to take medications (59.6%) and a video consultation with a doctor/nurse/midwife (59.0%). One-fourth of the surveyed nurses believe that video-consultations and research analysis applications are not worth recommending to patients. The pioneer of computerization in nursing is the United States, where various forms of care using an information system have been functioning successfully for many years. Some interesting initiatives in the field of digital health have also been carried out by Denmark, Finland, Sweden, and the United Kingdom. These include electronic medical records, the use of data in the clinical decision support system, expanding the groups of patients who can remotely use various forms of remote care [35]. The comprehensive legal solutions have been introduced in Germany (Digitale Versorgungs Gesetz) enabling the use of mobile applications in both diagnosis and treatment [36]. In Estonia, which is also a leader in the process of digitization, e-prescription data helps to assess whether a patient requires frequent consultations or hospitalization. In Sweden, on the other hand, medical innovation centres have been in operation for several years [37,38]. The above examples can be a source of inspiration and persuasive that the investment in digitization of healthcare is likely to bring measurable benefits, for both patients and medical staff [39,40,41]. In our study, nurses have opinions that the most useful e-health solutions are possibility to write out electronic sick leaves (46.5%), referrals (41.5%), easy and quick access to patients’ medical records (40.2%) and possibility to write electronic prescriptions (39.1%) declared that the most important e-health solutions were, first of all, the possibility of writing electronic sick leaves for patients (46.5%) referrals for medical exams (41.5%) and access to patients’ medical records (40.2%). Nurses’ usage of e-health solutions in their daily work ranges from very simple measuring devices to more sophisticated ones. Nurses are one of the first health workers to use e-health solutions for patient care [42,43]. According to the Canadian Nurses Association (CNA), telehealth nursing practice is an integrated part of healthcare that improves existing services by improving their accessibility, appropriate use, and efficiency [44]. As indicated Nejadshafiee et al. implementation e-health solutions are very useful also for care management in disaster and emergency [45].

The results showed the nurses’ belief that the functioning of the health care system in the near future will be very dependent on new technologies (70.9%) and most consider it very helpful (60.9%). A sort of catalyst for the process of digitization of the system of healthcare turned out to be a pandemic of COVID-19. The pandemic reorganized our lives, and the crucial point was health protection with the need to service patients without the need for personal contact with medical personnel [46]. The IT solutions, which in many medical entities were in the form of projects and plans, were immediately launched so that the medical services could be provided [47,48]. According to analysts so-called “digital health” has evolved and the interest in the e-solutions in the health sector is on the increase. According to the British Jupiter Research, the number of users of various types of wellness applications and digital therapeutics will increase rapidly. According to the report, in 2020, about 627 million people used health monitoring software, and it forecasts that there will be as many as 1.4 billion users by 2025 [49].

The results of the logistic stepwise regression and the analysis of the classification tree model to which all independent variables were taken into account in a linear regression were included showed that the factors that had an impact on both IT competence of nurses related to their skills and the need for training were level of education, additional qualifications and the workplace of the respondents. The lower the level of education of the nurses, without additional qualifications and older and working in PHC, the lower the IT competence in relation of skills and the need for training in this area than their younger colleagues with higher education, additional qualifications and working in a hospital. The obtained results may result from the average age of currently working nurses, which in Poland is 53 years. It is a generation that has learned basic IT skills only in adulthood, which makes it much more difficult to acquire e-competency [3,6]. Similarly, the authors of a study conducted in Canada on the factors related to the IT competence of nurses say that age, education, workplace, and attitude to lifelong learning determine the IT competence of nurses and the attitude to use modern technologies in everyday work. Moreover, the self-assessment of IT competence declared by the nurses does not always correspond to the actual competence in this area. The quality of IT training and the support offered by the employers had the greatest impact on the diversification of IT the competence results [28,29].

The research shows that the rapid development of e-Health increased the demand for nurses with high IT competence in healthcare entities, and thus the need to organize courses/trainings that could significantly increase the IT competence of nurses [50]. The regression results of a cross-sectional study in Seoul, Korea show that basic electronic equipment skills and the incorporation of computer science into formal nursing programmes are needed to improve nurses’ competence in managing and using health information [28].

This study has some limitations, as it is a cross-sectional study making use of a proprietary questionnaire. Moreover, the IT competence of nurses were not verified, it was the nurses’ self-assessment of their IT competence. Another limitation regarding this study is the time of the research, just before the COVID-19 pandemic, when no one realized how useful it would be to use the new technologies in patient care, which may explain the attitude of the surveyed nurses to the use of the new technologies in patient care. There are plans to carry out the study again, to compare how the attitudes of nurses using new technologies in their daily work have changed and to measure the IT competence with the use of specialized questionnaires.

## 5. Conclusions

Despite the fact that nurses assessed their IT competence as good enough, the need for training in this area was indicated. They most often recommend obtaining laboratory test results and arranging medical appointments via the Internet in their daily work. Nurses considered that having the ability to write electronic sick leaves, make electronic referrals, and to obtain easy/quick access to patients’ medical records in electronic form as the most important e-health solutions. Nurses find the use of new technologies in their daily life and at work very helpful. Additionally, the factors significantly affecting the IT competence of nurses were the level of education, additional qualifications, and the workplace. Younger nurses with a higher level of education and additional qualifications assessed higher the use of e-health solutions and their recommendations. The need for training in IT competence was higher in nurses working in the hospital. Nurses’ IT competencies both in relation to skills and the need for training are influenced by significant variables, such as assessment of the influence of the Internet and new technologies on the health care and lives of modern people, assessment of e-health solutions, recommendations of e-health solutions and age of respondents. The results of the study can be used to improve the IT competence of nurses and support the health care system. In addition, they provide the basis for appropriate policies to meet computer science education and support the needs of current and future nurses.

## Figures and Tables

**Table 1:** Intraclass correlation coefficient calculated for each type of questionnaire section.

Type of Section	ICC	96% CI (ICC)	
IT competence	0.448	0.387	0.504
Assessment of the influence of the Internet and new technologies on health care and modern life	0.279	0.020	0.459
Recommendation of e-health solutions	0.838	0.815	0.858
Assessment of e-health solutions	0.883	0.867	0.897

ICC—intraclass correlation coefficient, CI—confidence interval.

**Table 2:** The frequency of using electronic devices by nurses in private life and at work.

Type of Electronic Device	Often		Sometimes		Never		Not Appplicable	
	N	%	N	%	N	%	N	%
**In private life**								
Computer	534	62.9	282	33.2	31	3.7	2	0.2
Tablet	115	13.5	206	24.3	384	45.2	144	17.0
Smartphone	694	81.7	65	7.7	75	8.8	15	1.8
E-mail	392	46.2	333	39.2	108	12.7	16	1.9
Mobile applications	355	41.8	265	31.2	185	21.8	44	5.2
**At work**								
Computer	614	72.3	140	16.5	64	7.5	31	3.7
Tablet	25	2.9	41	4.8	557	65.6	226	26.6
Smartphone	226	26.6	235	27.7	286	33.7	102	12.0
E-mail	184	21.7	216	25.4	337	39.7	112	13.2
Mobile applications	84	9.9	157	18.5	439	51.7	169	19.9

**Table 3:** Nurses’ opinion on IT competence and the education in this field.

Item	Yes		Rather Yes		I Do Not Have Opinion		Rather No		No	
	N	%	N	%	N	%	N	%	41	4.8
I feel prepared to use e-health solutions in my work	240	28.3	369	43.5	153	18.0	46	5.4	69	8.1
I would use training courses to improve my IT competence	430	50.6	243	28.6	94	11.1	13	1.5	112	13.2
Current nursing education keeps pace with the challenges of the 21st century.	271	31.9	199	23.4	213	25.1	54	6.4	51	6.0
The nursing training program should better prepare for acquiring IT competence	354	41.7	186	21.9	230	27.1	28	3.3	41	4.8

**Table 4:** Types of e-health solutions recommended by nurses.

Recommended e-Health Solutions	I Recommend Now		I Would Recommend		I Do Not Recommend	
	N	%	N	%	N	%
Remote monitoring of basic parameters (pressure, heart rate, temperature, glucose level).	311	36.6	434	51.1	104	12.2
Laboratory test results via the Internet	390	45.9	396	46.6	63	7.4
Arranging medical appointments via the Internet	352	41.5	414	48.8	83	9.8
Using a mobile application that facilitates research analysis	210	24.7	458	53.9	181	21.3
Using a mobile application that is a knowledge base on health-related topics	209	24.6	507	59.7	133	15.7
Using a mobile application that is a mobile drug database	211	24.9	476	56.1	162	19.1
Using a mobile application that reminds to take medication	272	32.0	506	59.6	71	8.4
Using video-consultation with a doctor/nurse to support the treatment process	136	16.0	501	59.0	212	25.0

**Table 5:** Nurses’ opinions on the use of e-health solutions in the health care sector.

Types of e-Health Solutions	Strongly Relevant		Relevant		I Have No Opinion		Irrelevant		Completely Irrelevant	
	N	%	N	%	N	%	N	%	N	%
Easy and quick access to patients’ medical records	341	40.2	404	47.6	86	10.1	18	2.1	0	0.0
Possibility to write electronic prescriptions	332	39.1	413	48.6	93	11.0	8	0.9	3	0.4
Possibility to write out electronic sick leaves	395	46.5	399	47.0	43	5.1	8	0.9	4	0.5
Possibility to write electronic referrals	352	41.5	416	49.0	71	8.4	6	0.7	4	0.5
Using the electronic database of drugs	290	34.2	416	49.0	121	14.3	17	2.0	5	0.6
Ability to remotely route patients to other specialists or hospitals	269	31.7	453	53.4	112	13.2	8	0.9	7	0.8
Solutions to streamline the sending/sharing of clinical results	323	38.0	406	47.8	100	11.8	9	1.1	11	1.3
Solutions enabling remote (not requiring direct contact) patient care	166	19.6	397	46.8	243	28.6	34	4.0	9	1.1
Increasing the share of digital solutions supporting the treatment and self-monitoring of the patient’s health	181	21.3	429	50.5	211	24.9	20	2.4	8	0.9
The ability to exercise comprehensive control over the facility and track generated costs, staff management (schedules, schedules)	214	25.2	383	45.1	207	24.4	29	3.4	16	1.9
Possibility to conduct scientific research	219	25.8	371	43.7	219	25.8	30	3.5	10	1.2

**Table 6:** Nurses’ opinions on the presence of new technologies in the lives of modern people.

Type of Opinion	N	%	% Cumulated
It fascinates me	28	3.3	3.3
It interests me	188	22.1	25.4
It is helpful	517	60.9	86.3
I have no opinion	70	8.2	94.6
It worries me	31	3.7	98.2
It scares me	15	1.8	100.0

**Table 7:** Nurses’ IT competence in relation to skills depending on selected variables (assessment of the influence of the Internet and new technologies on the health care and lives of modern people, assessment of the e-health solutions, recommendations of e-health solutions and age of respondents).

Variables			N-SC		SC	t	*p*
			B	SE	β		
IT competence as skills		Assessment of the influence of the Internet and new technologies on health care and lives of modern people	0.203	0.034	0.203	5.941	0.0000
		Recommendation of e-health solutions	0.175	0.035	0.175	5.015	0.0000
		Assessment of e-health solutions	0.149	0.035	0.149	4.256	0.0000
		Age	0.008	0.003	0.095	2.952	0.0032
IT competence as skills	SME	Assessment of e-health solutions	0.184	0.062	0.194	2.989	0.0031
		Recommendation of e-health solutions	0.198	0.060	0.198	3.311	0.0011
		Assessment of the influence of the Internet and new technologies on health care and lives of modern people	0.168	0.061	0.172	2.766	0.0061
		Age	0.016	0.007	0.119	2.178	0.0303
	BD	Assessment of e-health solutions	0.210	0.053	0.210	3.965	0.0001
		Recommendation of e-health solutions	0.145	0.055	0.145	2.657	0.0083
		Assessment of the influence of the Internet and new technologies on health care and lives of modern people	0.164	0.053	0.164	3.092	0.0022
		Age	0.011	0.004	0.135	2.667	0.0080
	MD	Assessment of e-health solutions	0.267	0.064	0.267	4.169	0.0000
		Recommendation of e-health solutions	0.175	0.066	0.170	2.652	0.0086
IT competence as skills	No AQ	Assessment of e-health solutions	0.198	0.036	0.199	5.432	0.0000
		Recommendation of e-health solutions	0.172	0.037	0.172	4.719	0.0000
		Assessment of the influence of the Internet and new technologies on health care and lives of modern people	0.187	0.036	0.189	5.254	0.0000
		Age	0.008	0.003	0.095	2.794	0.0053
	AQ	Assessment of the influence of the Internet and new technologies on health care and lives of modern people	0.283	0.106	0.259	2.671	0.0088
IT competence as skills	H	Assessment of the influence of the Internet and new technologies on health care and lives of modern people	0.230	0.056	0.227	4.099	0.0001
		Recommendation of e-health solutions	0.188	0.055	0.192	3.417	0.0007
		Assessment of e-health solutions	0.183	0.058	0.172	3.138	0.0019
	PHC	Assessment of the influence of the Internet and new technologies on health care and lives of modern people	0.187	0.043	0.189	4.299	0.0000
		Recommendation of e-health solutions	0.171	0.046	0.165	3.742	0.0002
		Age	0.011	0.003	0.128	3.169	0.0016
		Assessment of e-health solutions	0.132	0.045	0.137	2.944	0.0034

SME—secondary medical education; BD—bachelor’s degree; MD—master’s degree; No AQ—no additional qualifications; AQ—possessing additional qualifications, PHC—primary health care; H—hospital; N-SC—non-standardized coefficients, SC—standardized coefficients; SE—standard error; B—regression coefficient; β—standardized regression coefficient; t—Student t-test; *p*—significance of regression coefficient.

**Table 8:** Nurses’ IT competence in relation the need for training depending on selected variables (assessment of the influence of the Internet and new technologies on health care and lives of modern people, assessment of the e-health solutions (generally and in relation to medical facility, recommendations of e-health solutions and age of respondents).

Variables			N-SC		SC	t	*p*
			B	SE	β		
IT competence as the need for training		Age	0.032	0.003	0.392	12.405	0.0000
		Assessment of e-health solutions (in realtion to medical facility)	0.074	0.032	0.074	2.344	0.0193
IT competence as the need for training	SME	Assessment of the influence of the Internet and new technologies on health care and lives of modern people	0.037	0.007	0.293	5.065	0.0000
	BD	Age	0.040	0.004	0.447	9.131	0.0000
		Assessment of e-health solutions	−0.130	0.053	−0.119	−2.440	0.0152
	MD	Age	0.020	0.005	0.235	3.693	0.0003
		Assessment of e-health solutions (in realtion to medical facility)	0.172	0.062	0.177	2.778	0.0059
IT competence as the need for training	No AQ	Age	0.034	0.003	0.411	12.323	0.0000
	AQ	Assessment of e-health solutions (in realtion to medical facility)	0.212	0.075	0.268	2.845	0.0054
		Age	0.027	0.009	0.284	3.056	0.0029
		Recommendation of e-health solutions	−0.265	0.090	−0.318	−2.942	0.0041
		Assessment of e-health solutions	0.215	0.092	0.241	2.336	0.0216
IT competence as the need for training	H	Age	0.028	0.005	0.325	6.005	0.0000
	PHC	Age	0.032	0.003	0.391	9.943	0.0000
		Assessment of e-health solutions (in realtion to medical facility)	0.117	0.037	0.126	3.199	0.0015

SME—secondary medical education; BD—bachelor’s degree; MD—master’s degree; No AQ—no additional qualifications; AQ—possessing additional qualifications, PHC—Primary Health Care; H—Hospital; N-SC—Non-Standardized Coefficients, SC—Standardized Coefficients; SE—standard error; B—regression coefficient; β—standardized regression coefficient; t—Student’s t-test; *p*—significance of regression coefficient.

## Data Availability

The data presented in this study are available on reasonable request from the corresponding author. The data are not publicly available due to restrictions, e.g., their containing information that could compromise the privacy of research participants.

## Supplementary Materials

The following are available online at https://www.mdpi.com/article/10.3390/jcm10204799/s1, File S1: Survey questionnaire. File S2: Classification tree, CHAID method—Figures S1 and S2.
