# Supplementary material for: Polish Nurses’ Attitude to e-Health Solutions and Self-Assessment of Their IT Competence
Source: J Clin Med. 2021 Oct 19;10(20):4799. doi: 10.3390/jcm10204799 (PMC8540281; doi:10.3390/jcm10204799)
Supplement: Supplementary file 1 [file jcm-10-04799-s001.zip › CHAID_method_fig.S1 and fig.S2.pdf]

Classification tree, CHAID method- Figure S1 and Figure S2.

CHAID analysis
In the case of IT competences related to the need for training, 8 nodes were distinguished. The first 4 nodes were included in the so-called age variable, with nodes 1 and 2 containing low mean values of training needs (mean <0) and these were nurses up to 24 years of age and 24-37 years of age. The next two nodes were defined by the age groups 37-56 and over 56. In these nodes, the IT competences related to the need for training were bigger (average> 0), therefore for this group of nurses was a stronger need for training. The next two nodes (5 and 6) were defined by having additional qualifications. The nurses with additional qualifications needed training to a lesser extent to increase their IT competences (average <0), while nurses with no additional qualifications obtained a higher index corresponding to the need to conduct training (average> 0). The last two nodes were built based on the workplace of the surveyed nurses. A stronger need for training in term of IT competences was found in nurses working in PHC and other health care entities (PS, OSC) (average 0.291) than in the nurses employed in a hospital (average 0.068).
The nurses' IT competences in question to skills were defined by means of 12 nodes. The variables that significantly influenced the determination of the nurses' IT competences were the assessment of the impact of the Internet on health care, recommendation of the e-health solutions and the assessment of the e-health solutions for the patient. The IT competences related to skills were mainly determined by node 2 containing 59.5% of the observations. In each of the nodes, the range of the nurses’ IT competences related to the skills of nurses were defined. Node 1 consisted of people who obtained the level of assessment of the impact of the Internet and new technologies on the health care and life of modern people not exceeding the value of -0.684003. At this node, the IT competency values related to the skills were low (mean -0.501). Thus, this node corresponded to those who rated the impact of the Internet on healthcare higher, which in turn translated into higher IT competences related to skills. Node 2 consisted of people with an average assessment of the impact of the Internet on health care (range -0.684003-0.642527) - these nurses obtained a competence index of 0.024. Node 3 were those with Internet impact assessment scores in excess of 0.642527, which generated a skills-related digital literacy level of 0.587.

In order to make a presentation of the obtained results more visible, a classification tree was used namely the CHAID method, which consists of grouping the variables that significantly determined the level of the nurses’ IT competences, separately regarding their skills and the need for training. The classification tree model includes all independent variables taken into account in linear regression (workplace, specialization, education and quantitative variables: assessments of e-health solutions, recommendation of e-health solutions, assessment of the impact of the Internet on health care). The tree was built with the use of the CHAID method that extracts only significant variables, the quantitative variables were grouped into compartments that build a given node (i.e. the variable was divided into parts and checked at what values of the independent variable the data for the value of the dependent variable are obtained (which, in relation to the presented results, concerned the level of competences of nurses) In the case of a nominal variable (eg education), each category is analyzed separately.
In the case of IT competences related to the need for training, 8 nodes were distinguished. The first 4 nodes were included in the so-called age variable, with nodes 1 and 2 containing low mean values of training needs (mean <0) and these were nurses up to 24 years of age and 24-37 years of age. The next two nodes were defined by the age groups 37-56 and over 56. In these nodes, the IT competences related to the need for training were bigger (average> 0), therefore for this group of nurses was a stronger need for training. The next two nodes (5 and 6) were defined by having additional qualifications. The nurses with additional qualifications needed training to a lesser extent to increase their IT competences (average <0), while nurses with no additional qualifications obtained a higher index corresponding to the need to conduct training (average> 0). The last two nodes were built based on the workplace of the surveyed nurses. A stronger need for training in term of IT competences was found in nurses working in PHC and other health care entities (PS, OSC) (average 0.291) than in the nurses employed in a hospital (average 0.068).
(Figure S1).

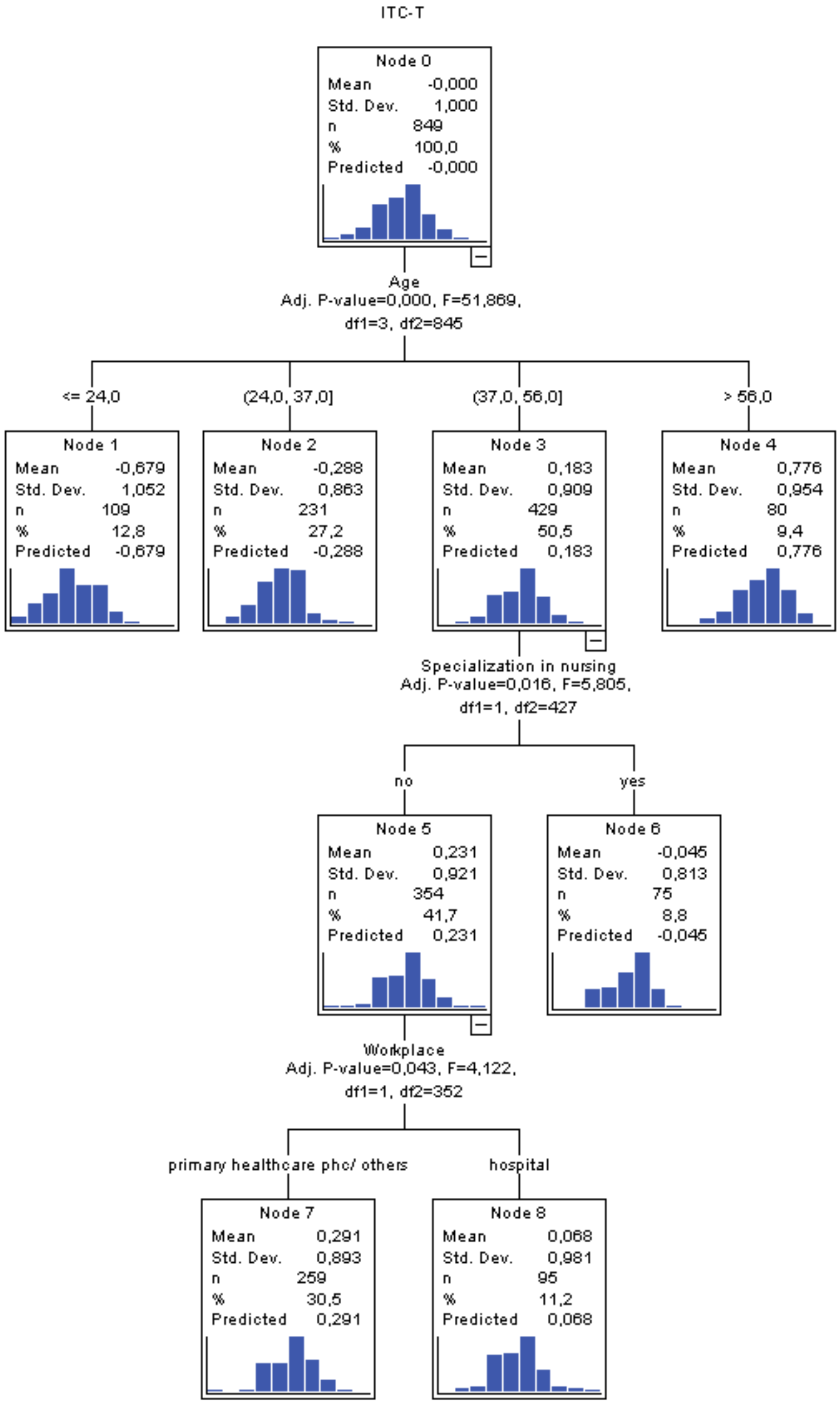
ITC-T
Node 0
Mean -0,000
Std. Dev. 1,000
n 849
% 100,0
Predicted -0,000
Age
Adj. P-value=0,000, F=51,869,
df1=3, df2=845
<= 24,0
(24,0, 37,0]
(37,0, 56,0]
> 56,0
Node 1
Mean -0,679
Std. Dev. 1,052
n 109
% 12,8
Predicted -0,679
Node 2
Mean -0,288
Std. Dev. 0,863
n 231
% 27,2
Predicted -0,288
Node 3
Mean 0,183
Std. Dev. 0,909
n 429
% 50,5
Predicted 0,183
Node 4
Mean 0,776
Std. Dev. 0,954
n 80
% 9,4
Predicted 0,776
Specialization in nursing
Adj. P-value=0,016, F=5,805,
df1=1, df2=427
no
yes
Node 5
Mean 0,231
Std. Dev. 0,921
n 354
% 41,7
Predicted 0,231
Node 6
Mean -0,045
Std. Dev. 0,813
n 75
% 8,8
Predicted -0,045
Workplace
Adj. P-value=0,043, F=4,122,
df1=1, df2=352
primary healthcare phc/ others
hospital
Node 7
Mean 0,291
Std. Dev. 0,893
n 259
% 30,5
Predicted 0,291
Node 8
Mean 0,068
Std. Dev. 0,981
n 95
% 11,2
Predicted 0,068

ITC - IT competences related to training

df - degrees of freedom (two measures of df were counted, hence df1 and df2)

F - the value of the F-Snedecor test

AM- arithmetic means

SD – standard deviation

n- sample size

Figure S1. Nurses' IT competences related to training (the higher the results, the higher the need for training).

The nurses' IT competences in question to skills were defined by means of 12 nodes. The variables that significantly influenced the determination of the nurses' IT competences were the assessment of the impact of the Internet on health care, recommendation of the e-health solutions and the assessment of the e-health solutions for the patient. The IT competences related to skills were mainly determined by node 2 containing 59.5% of the observations. In each of the nodes, the range of the nurses' IT competences related to the skills of nurses were defined. Node 1 consisted of people who obtained the level of assessment of the impact of the Internet and new technologies on the health care and life of modern people not exceeding the value of -0.684003. At this node, the IT competency values related to the skills were low (mean -0.501). Thus, this node corresponded to those who rated the impact of the Internet on healthcare higher, which in turn translated into higher IT competences related to skills. Node 2 consisted of people with an average assessment of the impact of the Internet on health care (range -0.684003-0.642527) - these nurses obtained a competence index of 0.024. Node 3 were those with Internet impact assessment scores in excess of 0.642527, which generated a skills-related digital literacy level of 0.587. (Figure S2).

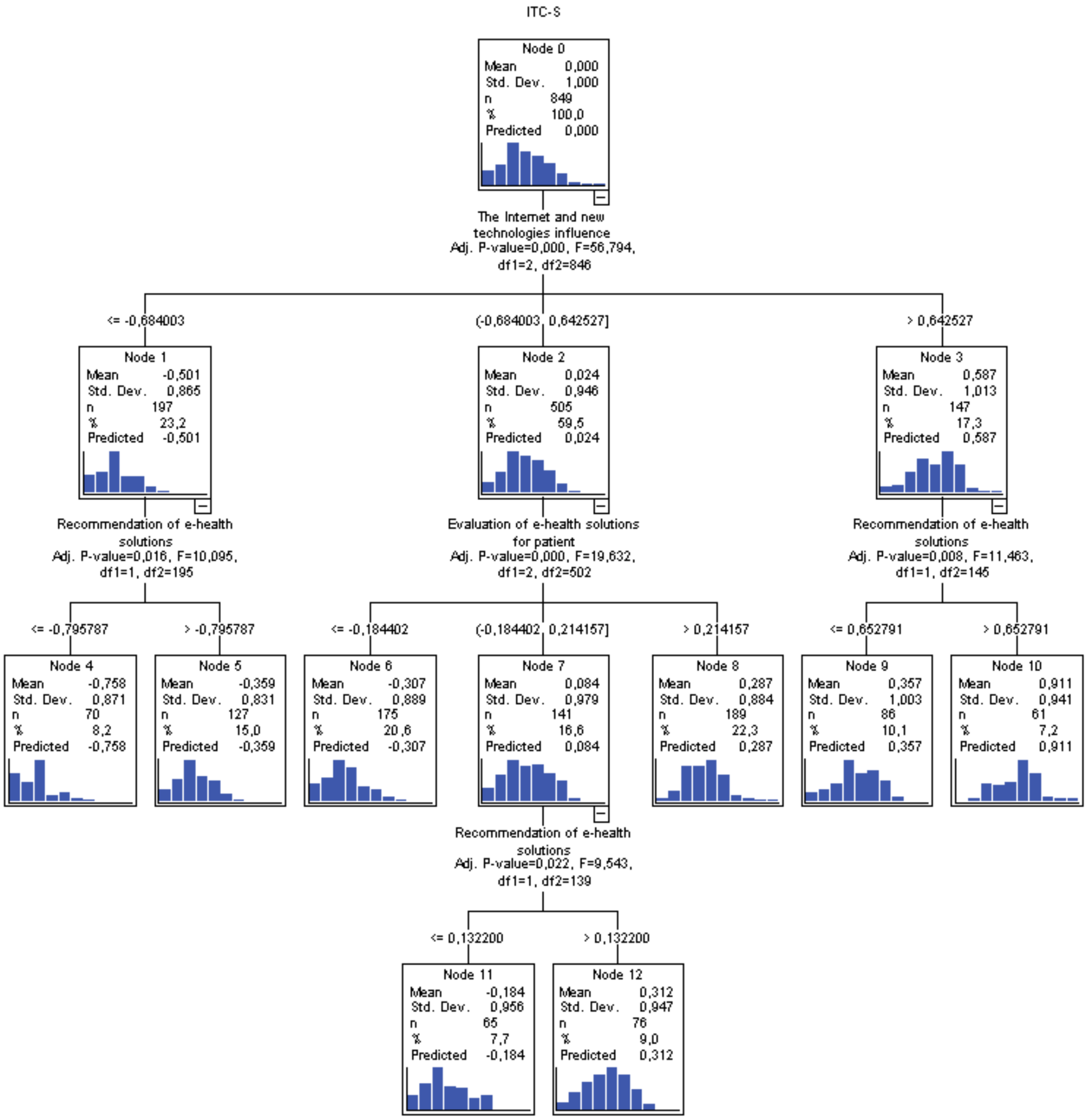

ITC-S - IT competences related to skills

df - degrees of freedom (two measures of df were counted, hence df1 and df2)

F - the value of the F-Snedecor test

AM- arithmetic means

SD – standard deviation

n- sample size

Figure S2.Nurses' IT competences related skills (the higher the results, the lower the skills).
