# Supplementary material for: Polish Nurses’ Attitude to e-Health Solutions and Self-Assessment of Their IT Competence
Source: J Clin Med. 2021 Oct 19;10(20):4799. doi: 10.3390/jcm10204799 (PMC8540281; doi:10.3390/jcm10204799)
Supplement: Supplementary file 1 [file jcm-10-04799-s001.zip › Survey questionnaire.pdf]

Survey questionnaire

Nurses' Attitude to E-health Solutions and Self-assessment of their IT Competences

Socio-demogrphic data of survey nurses:

Age…………………………..

Level of education:

- ☐ Medical secondary education
- ☐ Bechelor's degree
- ☐ Master's degree

Additional qualification

☐ Yes

☐ No

Place of work

- ☐ Hospital
- ☐ Primary Health Care
- ☐ Private sector
- ☐ Outpatient Specialist Care

The frequency of using the internet and the electronic devices by nurses in private life and at work

How often do you use the Internet?

- ☐ Several times a day
- ☐ Every day
- ☐ Several times a week
- ☐ Once a week
- ☐ I don't use it

	**In private life**				**At work**				
	Often	Sometimes	Never	Not applicable		Often	Sometimes	Never	Not applicable
Computer					Computer				

Tablet					Tablet				
Smartphone					Smartphone				
E-mail					E-mail				
Mobile Apps					Mobile Apps				

IT competences - Nurses' opinion on IT competences and the education in this field

Please evaluate your skills in using these devices / solutions and the Internet:

- ☐ Very good
- ☐ Good
- ☐ Sufficient
- ☐ Weak
- ☐ Very weak

Item	Yes	Rather yes	I do not have opinion	Rather no	No
I feel prepared to use e-health solutions in my work					
I would use training courses to improve my IT competences					
Current nursing education keeps pace with the challenges of the 21st century.					
The nursing training program should better prepare for acquiring IT competences					

Recommendation of e-health solution section

Types of e-health solutions recommended by nurses:

Recommended e-health solutions	I recommend now	I would recommend	I do not recommend
Remote monitoring of basic parameters (pressure, heart rate, temperature, glucose level).			
Laboratory test results via the Internet			
Arranging medical appointments via the Internet			
Using a mobile application that facilitates research analysis			
Using a mobile application that is a knowledge base on health-related topics			

Using a mobile application that is a mobile drug database			
Using a mobile application that reminds to take medication			
Using video-consultation with a doctor/nurse to support the treatment process			

Assessment of e-health solutions section

Nurses' opinions on the use of e-health solutions in the health care sector:

Types of e-health solutions	Strongly relevant	Relevant	I have no opinion	Irrelevant	Completely irrelevant
Easy and quick access to patients' medical records					
Possibility to write electronic prescriptions					
Possibility to write out electronic sick leaves					
Possibility to write electronic referrals					
Using the electronic database of drugs					
Ability to remotely route patients to other specialists or hospitals					
Solutions to streamline the sending / sharing of clinical results					
Solutions enabling remote (not requiring direct contact) patient care					
Increasing the share of digital solutions supporting the treatment and self-monitoring of the patient's health					
The ability to exercise comprehensive control over the facility and track generated costs, staff management (schedules, schedules)					
Possibility to conduct scientific research					

Nurses' opinions on the presence of new technologies in the life of modern people

- ☐ It fascinate me
- ☐ It interest me
- ☐ It is helpfull
- ☐ I have no opinion
- ☐ It worries me
- ☐ It scares me

Thank you for participating in the survey
